# A novel, percutaneous, self-expanding, forceful reduction screw system for the treatment of thoracolumbar fracture with severe vertebral height loss

**DOI:** 10.1186/s13018-018-0880-4

**Published:** 2018-07-11

**Authors:** Qinpeng Zhao, Dingjun Hao, Biao Wang

**Affiliations:** Department of Spine Surgery, Honghui Hospital, Xi’an Jiaotong University College of Medicine, No. 76 Nanguo Road, Xi’an, 710054 Shaanxi China

**Keywords:** Thoracolumbar fracture, Minimal invasive surgery, Novel screw system

## Abstract

**Background:**

Over the past decade, the techniques for minimally invasive spinal stabilization have improved significantly. The multiaxial screw utilized in minimally invasive operations is limited in restoring fracture height, reconstructing the anterior vertebral column, and improving kyphosis. Therefore, the percutaneous, minimally invasive approach is not recommended for a thoracolumbar fracture with severe vertebral height loss. We report our novel, percutaneous, self-expanding, forceful reduction screw system to address this problem.

**Methods:**

Thirty-eight patients experiencing thoracolumbar fracture, with a vertebral height loss more than 50%, were treated with the novel, percutaneous, self-expanding, forceful reduction screw between March 2014 and June 2015. The patients’ charts and radiographs were reviewed. The vertebral body index (VBI), height of the anterior margin of fractured vertebra (HAMFV), vertebral body angle (VBA), bisegmental Cobb angle (BCA), and Oswestry disability index (ODI) scores were obtained before and after the operation, as well as during the 2-year follow-up. The scoring results were compared using *t* tests.

**Results:**

The operation was completed successfully in 38 patients. A total of 152 screws were placed. The average operation time was 90.7 ± 21.9 min, and the average intraoperative bleeding amount was 89.2 ± 31.9 ml. The patients were discharged at a mean of 3.2 ± 0.9 postoperative days, with a mean hospital stay of 4.8 ± 1.0 days. The VBI, HAMFV, VBA, and BCA scores were significantly improved after treatment with the novel screw system; there was a significant difference between pre- and postoperative parameters (*p* < 0.05). Although the decreases in all of the parameters were variable during the 2-year follow-up, there were no statistical differences between the postoperative imaging parameters and the last follow-up imaging parameters (*p* > 0.05). The ODI score at the last follow-up examination was 5.9 ± 2.7, which was significantly improved compared with the preoperative score of 44.6 ± 2.3 (*p* < 0.05).

**Conclusions:**

We believe that the novel, percutaneous, self-expanding, forceful reduction screw system developed by us not only successfully expands the minimally invasive percutaneous surgery to the thoracolumbar fracture with severe vertebral height loss but also achieves significant vertebral height restoration and kyphosis correction.

## Background

Spinal fractures account for approximately 5–6% of total fractures and are most commonly found in the thoracolumbar spine, which is where approximately 75% of all vertebral fractures occur [1–3]. Currently, the posterior pedicle screw fixation and reduction approach has been well-accepted as a mainstream therapeutic mode. The conventional open surgical approach is usually associated with a larger operational incision and more severe surgical injury, especially concerning the intraoperative extensive detachment and traction of paraspinal muscles and damage to the posterior branch of the spinal nerve, which would easily lead to postoperative muscle atrophy and scar formation, resulting in postoperative, chronic, intractable back pain and limited motion, severely affecting the functionality recovery and life quality of the patients [4–6].

Over the past decade, the techniques for minimally invasive spinal stabilization have improved significantly. The minimally invasive surgery approach to pedicle screw instrumentation of thoracolumbar fractures minimizes soft tissue injury, reduces intraoperative blood loss, and results in better postoperative pain scores than other approaches [7–10]. Though the minimally invasive approach could be utilized to avoid those postoperative complications, the multiaxial screw utilized in the minimally invasive operation has limitations in the restoration of fracture height, reconstruction of anterior vertebral column, and kyphosis improvement. Therefore, the percutaneous minimally invasive approach was not recommended for the thoracolumbar fracture with severe vertebral height loss (vertebral height loss > 50%); instead, the conventional open operation was suggested [7, 9, 11, 12].

A novel, percutaneous, self-expanding, forceful reduction screw system was developed by us with the objective of adopting the percutaneous minimally invasive approach for the treatment of thoracolumbar fracture with severe vertebral height loss. In this study, a total of 38 cases were retrospectively analyzed and reported, all of which were thoracolumbar fracture with severe vertebral height loss treated by our novel percutaneous fixation system.

## Methods

Thirty-eight patients with thoracolumbar fracture with vertebral height loss of more than 50% were treated with the novel, percutaneous, self-expanding, forceful reduction screw between March 2014 and June 2015, including 29 men and 16 women. The patients were 35.9 ± 9.0 years old (ranging between 17 and 58 years old). The fracture locations included T11 for 5 patients, T12 for 12 patients, L1 for 17 patients, and L2 for 4 patients. The causes of the fractures included traffic accidents for 17 patients, falling injuries for 15 patients, sports injuries for 4 patients, and injuries related to heavy objects crashing for 2 patients. The inclusion criteria were single-level fracture, vertebral height loss > 50%, age less than 60 years old, no neurological deficit, and fracture level between T10 and L2. The exclusion criteria were pathological or osteoporotic fracture (bone mineral density (BMD) of < 2.5 standard deviation below the age-corrected mean was defined as osteoporosis), multilevel fracture, or previous surgery at the site of fracture. After an explanation of the surgical options, all patients signed an informed consent form authorized by the local ethics committee. The patients’ demographic information is given in Table 1.

**Table 1:** Demographic and clinical characteristics of the patients

Characteristics	Value
Number of patients	38
Age (years)	35.9 ± 9.0 (range 17–58)
Male to female ratio	29:16
Fracture locations	
T11	5 patients
T12	12 patients
L1	17 patients
L2	4 patients
Surgical duration (minutes)	90.7 ± 21.9
Blood loss (milliliters)	89.2 ± 31.9
Postoperative stay (days)	3.2 ± 0.9
Hospital stay (days)	4.8 ± 1.0

**Fig. 1:**
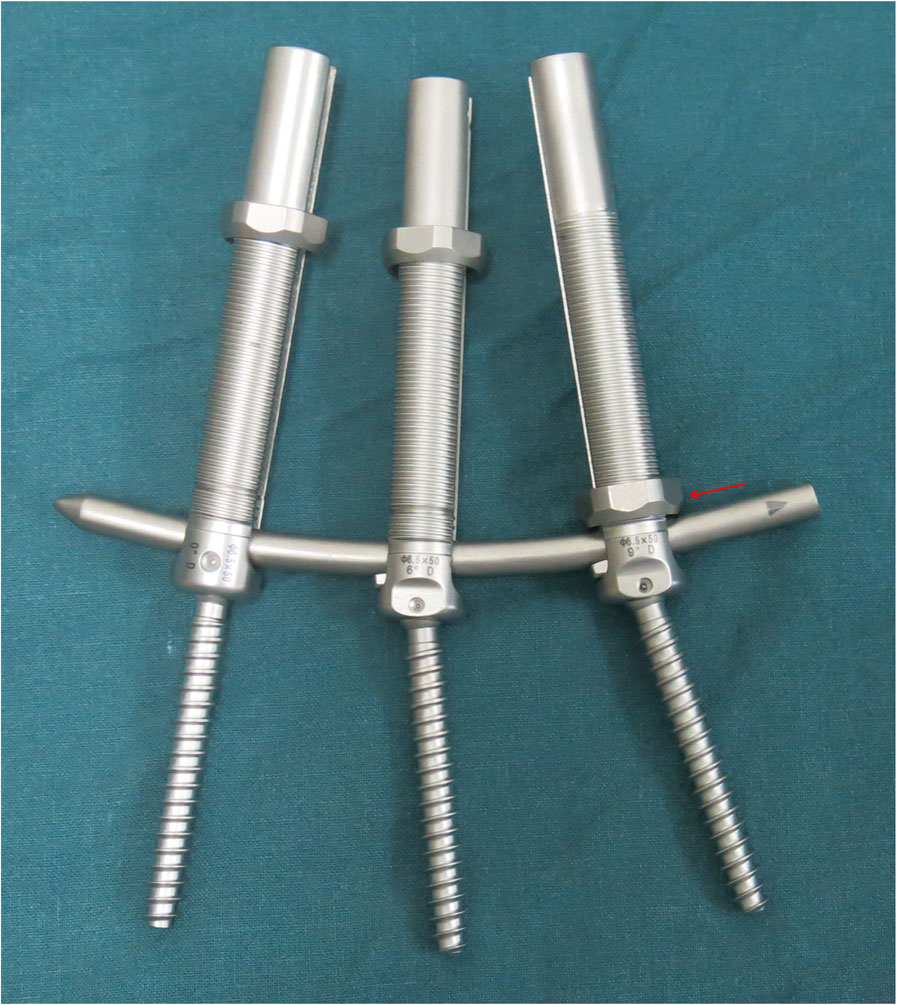
The novel percutaneous self-expanding forceful reduction screw system presented is uniaxial screws with reduction angles and drop-prevention bolts (red arrow). From the left to right screws, the reduction angles are 0°, 6°, and 9° in turn

All of the patients were treated with the percutaneous, minimally invasive reduction and fixation integrated with the novel, self-expanding, forceful, reduction screw system. The patients were placed in prone position after general anesthesia; the abdominal part was suspended by placement of cushions under the chest and anterior superior iliac spine. The thoracolumbar overextension facilitated the reduction of fracture. After postural reduction, the surface projections of the bilateral pedicle lateral margins of superior and inferior vertebra were marked under the visualization of C-arm X-ray. A longitudinal incision of 1.5 cm was made on the outside of the surface projection marker with a distance approximately 1 to 1.5 cm according to the girth of the patient; the subcutaneous tissue and lumbodorsal fascia were cut open gradually, layer-by-layer. The puncture needle was placed on the outer edge of the pedicle projections (10 o’clock position on the left side and 2 o’clock position on the right side), guided with anteroposterior X-ray; the needle was inserted through the pedicle toward the vertebral body with a tilt of 10°–15° parallel to the end plate and ending at the rear edge of the vertebral body under the guidance of lateral X-ray inspection. The needle point remained within the medial margin of the pedicle, which was confirmed by the anteroposterior X-ray (to avoid, as much as possible, mistakenly entering the spinal canal). Then, the needle was further inserted parallel to the end plate over the vertebral posterior margin approximately 0.5 to 1 cm, guided by lateral X-ray. Next, the guide wire was placed; meanwhile, injury of blood vessels and organs was avoided by carefully inserting guide wire at the appropriate positions. The other three guide wires were inserted as well, using the same approach. The muscle expansion cannulas were gradually placed through guide wires, and the screw path was expanded by tapping. The novel type of percutaneous screw with proper length and reduction angle (0°, 3°, 6°, or 9°) was screwed into the vertebral body (Fig. 1). Once the position of the screw was confirmed to be appropriate by C-arm X-ray, the fixation rods were placed into the screw tail grooves through subcutaneous and deep muscles. The fixation screws were expanded by distractor at the junction between screw extension rod and skin; meanwhile, the ends of the screw extension rods were moved inward to facilitate the reduction of the anterior part of the fractured vertebral body. The screw caps were slowly tightened under X-ray inspection (the reduction angle created by the screw restores the height of vertebral body during the cap tightening). When the vertebral height was restored satisfactorily, all the caps were tightened, the drop-prevention bolts were placed, and the screw extension rods were removed before the incision was closed.

Antibiotics were administered for 24 h after the operation. The patients were encouraged to engage in activities with the protection of a brace. All the patients were discharged when their situation was stabilized. The braces were suggested for them to wear for 4 to 6 weeks after discharge, and the patients started to do exercises to recover functionality under the guidance of a rehabilitation doctor. The internal fixation material could be removed around 18 to 24 months postoperation after CT confirmation of fracture healing.

**Fig. 2:**
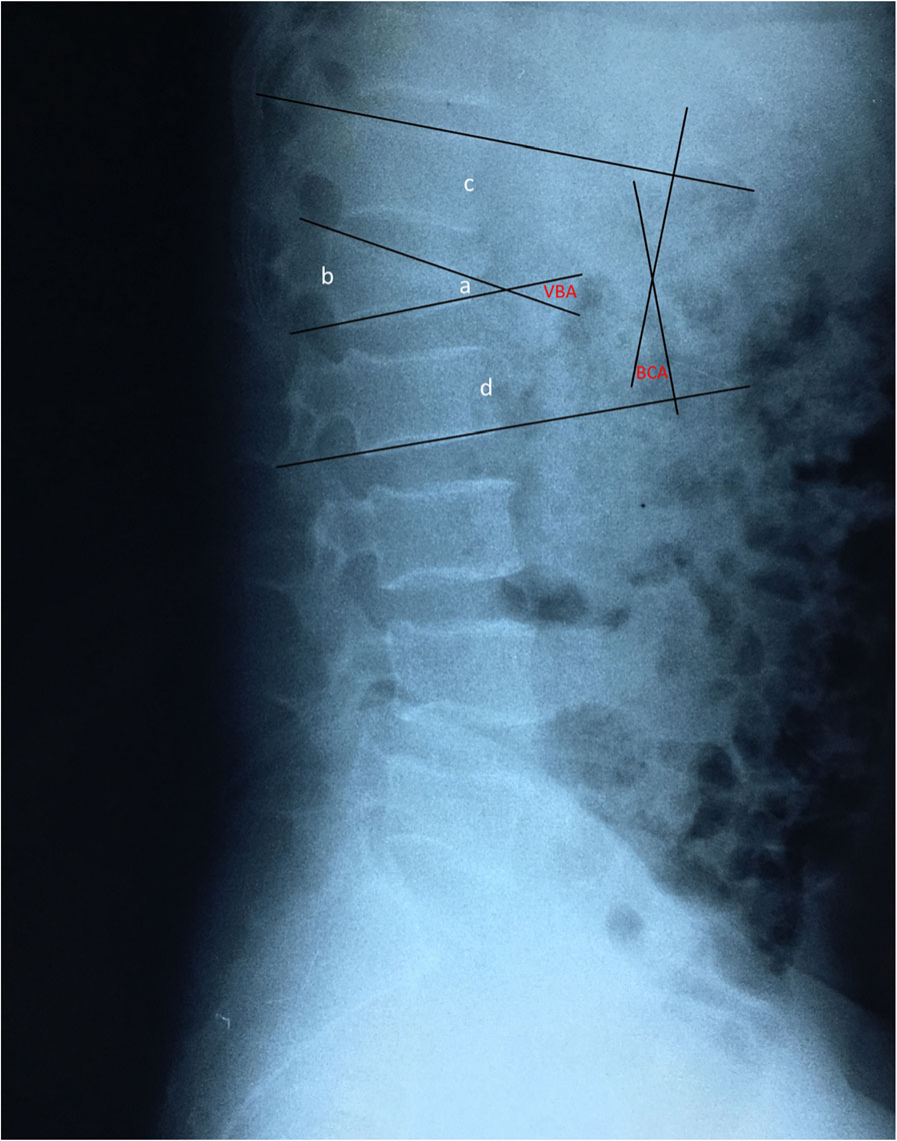
Illustration of the radiographic measurements: VBI = a/b; HAMFV = a/(c + d); VBA and BCA are as shown in this figure. A 51-year-old male with a fracture at L1 segment required surgical treatment. His preoperative radiographic parameters were as follows: VBI, 0.25; HAMFV, 0.27; VBA, 30°; and BCA, 21°

Radiographic evaluation consisted of supine admission radiography, CT, and MRI. The accuracy of screw placement was examined using CT after mobilization of each patient. Follow-up examinations at postoperative 3, 6, 9, 12, and 24 months were recommended; the fracture healing conditions were evaluated by X-ray and CT, to observe the position of internal fixation loosening, displacement, rupture, and kyphosis. Kyphotic deformity and radiographic follow-up were defined by the vertebral body index (VBI: relation between the anterior and posterior wall height of the fractured vertebra), height of the anterior margin of fractured vertebra (HAMFV: which is the percentage of the measured anterior margin height compared with the reference anterior margin height of the injured vertebrae; the reference height is the mean of the anterior margin height of the vertebral bodies adjacent to the injured one), vertebral body angle (VBA: angle defined by the upper and lower endplate of the fractured vertebra), and bisegmental Cobb angle (BCA: angle defined by the upper endplate of the first vertebra above the fractured one and by the lower endplate of the first vertebra below the fractured one) (Fig. 2). The patients’ recovery of functionality was evaluated with Oswestry disability index (ODI) scores.

The VBI, HAMFV, VBA, BCA, and ODI scores were obtained before and after the operation, as well as during the 2-year follow-up. They were compared by *t* test and statistically analyzed with SPSS 19.0 software (Chicago, IL, USA). The data are presented as the mean ± standard deviation, and *p* < 0.05 was considered statistically significant.

## Results

The operation was completed successfully in 38 patients. A total of 152 screws were placed. The average operation time was 90.7 ± 21.9 min, and the average intraoperative bleeding amount was 89.2 ± 31.9 ml. There were no complications as significant as large blood vessel injury or spinal cord injury. Incision wound fat liquefaction and delayed healing were reported by one patient with obesity; the incision was treated with a clean dressing and symptomatic treatment, and the incision wound was healed at postoperative day 22. There was no incision infection or hematoma detected. The patients were discharged at a mean of 3.2 ± 0.9 postoperative days, with a mean hospital stay of 4.8 ± 1.0 days.

**Fig. 3:**
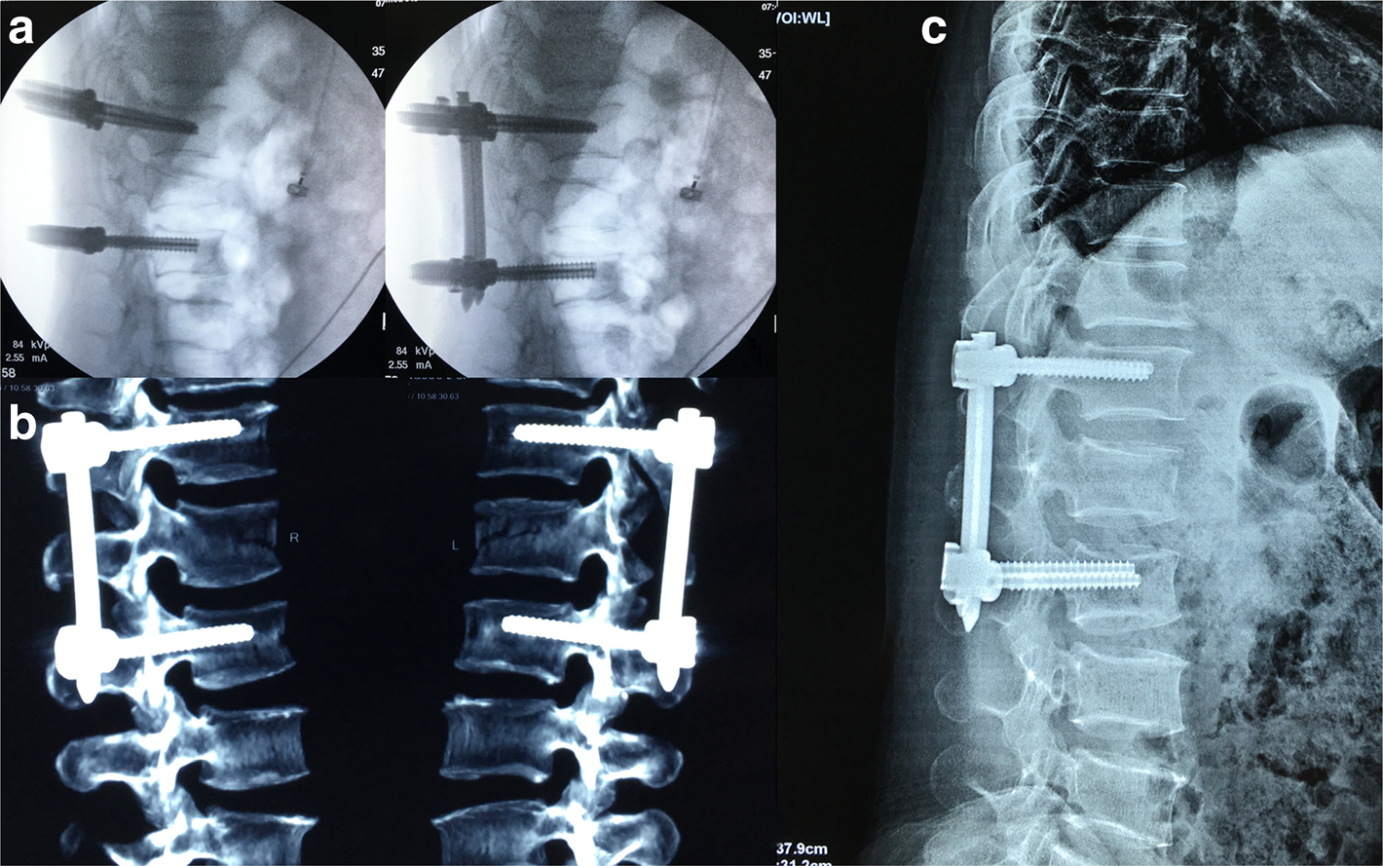
**a** The intraoperative contrast chart shows the vertebral height restoration and kyphosis correction by using the new screw system. **b** CT examination 6 months after the operation shows that the internal fixation position was good. **c** X-ray examination 2 years after surgery shows the reduction in vertebral height, and physiological curvature of the thoracolumbar spine was maintained well. His follow-up radiographic parameters were as follows: VBI, 0.95; HAMFV, 0.97; VBA, 2°; and BCA, 5°

On the postoperative CT scan, only one patient with a slim pedicle presented with the pedicle medial wall ruptured by two screws and a mild intrusion of spinal canal. However, there were no symptoms of neurological deterioration. The other 150 screws were properly placed, and the accuracy was 98.7%. All 38 patients were followed up for more than 2 years. There were no complications reported such as internal fixation loosening, displacement, or rupture. The internal fixation materials were removed from all the patients at the last follow-up examination. The imaging measurements were obtained before and after the operation as well as at the last follow-up examination. It was found that VBI, HAMFV, VBA, and BCA were significantly improved after treatment with the novel screw system, and there was a significant difference before and after the operation (*p* < 0.05). The improvement indicated that the novel screw system could restore vertebral height and correct kyphosis deformity. Although the decreases in all of the parameters were variable during the 2-year follow-up, there was no statistical significance between the postoperative imaging parameters and the last follow-up imaging parameters (*p* > 0.05), indicating that vertebral height and thoracolumbar curvature were well sustained. The ODI score at the last follow-up examination was 5.9 ± 2.7, which was significantly improved compared with the preoperative score of 44.6 ± 2.3 (*p* < 0.05). The detailed statistical information is shown in Table 2. A typical case is shown in Figs. 2 and 3.

**Table 2:** Statistical results of the radiographic parameters and ODI score

	Preoperative	Postoperative	Final follow-up	*P*_1_	*P*_2_	*P*_3_
VBI	0.38 ± 0.07	0.93 ± 0.03	0.92 ± 0.03	0.000	0.000	0.085
HAMFV	0.38 ± 0.06	0.95 ± 0.02	0.94 ± 0.02	0.000	0.000	0.107
VBA (°)	25.4 ± 4.5	3.7 ± 1.4	3.9 ± 1.3	0.000	0.000	0.090
BCA (°)	18.7 ± 8.6	5.8 ± 6.8	6.1 ± 7.0	0.000	0.000	0.118
ODI score	44.6 ± 2.3	24.4 ± 1.9	5.9 ± 2.7	0.000	0.000	0.000

Data are presented as the mean ± standard deviation

*ODI score* Oswestry disability index score, *P*_*1*_ postoperative compared with preoperative, *P*_*2*_ final follow-up compared with preoperative, *P*_*3*_ final follow-up compared with postoperative

## Discussion

Spine vertebral fractures are a common cause of pain and disability. Conservative management, including bed rest, pain relievers, bracing, and physical therapy, may fail to relieve pain and frequently lead to prolonged immobilization, which is associated with a negative impact on life quality and increased healthcare cost for the patient. In addition, although folk natural products studies provide a potentially promising way for treating bone loss diseases, which may be beneficial for the healing of fractures, but it has “long way to go” for clinical practice [13–16].

In the past decade, there has been an apparent trend to minimize soft tissue injury during spinal surgery. The concept of percutaneous targeting and access to the thoracic and lumbar pedicles was first described in 1984 by Magerl [17]. The technique did not provide the desired results and was therefore abandoned [18]. Foley et al. [19] described the first clinical series of a percutaneous technique for fusion of degenerative lumbar pathologies using a unique fixation system (Sextant, Medtronic, USA) in 2001. Three years later, Assaker [20] first reported on the application of percutaneous transpedicular fixation in thoracolumbar trauma, which was the initiation of arguments on the selection of a percutaneous minimally invasive approach or conventional open-surgical approach for the treatment of thoracolumbar fracture.

Patients with neurological deficit and spinal canal invasion by bone or soft-tissue fragments may require a surgical decompression as a rule. There is no doubt that most clinicians would such as to choose an open surgical approach. However, for patients without neurological symptoms but with spinal canal invasion, canal decompression is not mandatory. Indirect posterior decompression by means of distraction and lordosis forces can be very effective in obtaining a closed decompression in the thoracolumbar spine [21]. Both the conventional open approach and percutaneous minimally invasive approach could be considered for these patients.

A number of studies have been published on the comparison of standard open surgery and minimally invasive percutaneous surgery in thoracolumbar trauma. Most of the published reports [5, 6, 22], including four systematic reviews with high-quality evidence, such as Phan et al. [23], Koreckij et al. [12], McAnany et al. [24], and Sun et al. [25], indicated that there was no significant difference with respect to clinical efficacy between the two modes of surgery for the treatment of thoracolumbar fracture (type A fracture of Magerl classification with mild compression). Moreover, the minimally invasive surgery seemed to be preferred because of the shorter operation time, less intraoperative and postoperative bleeding, no need for blood transfusion, reduced hospital stay, decreased infection rate, better muscle protection, and faster recovery of functionality in addition to better efficacy. However, the percutaneous transpedicular screw system adopted in clinical practice was developed for the treatment of degenerative diseases; the fracture reduction capability was inadequate and not able to restore the vertebral height [9, 11, 12]. It was reported by Jiang et al. [7], which was the only randomized study with high-quality of evidence, that the minimally invasive approach was not suitable for the patients with postural reduction failure, and a more open approach may be desirable to achieve improved fracture reduction and deformity correction. Some studies proposed that the thoracolumbar fracture with clear need of reduction was the contraindication of percutaneous minimally invasive surgery [23]. Apparently, thoracolumbar fracture with vertebral height loss > 50% is not suitable for minimally invasive surgery since it theoretically cannot achieve adequate reduction and improve the kyphosis; thus, the open surgery should be considered.

Should the minimally invasive approach be given up in favor of open surgery for those patients with thoracolumbar fracture with vertebral height loss more than 50%? Is there any option that could enable the minimally invasive approach to be utilized for these patients? To address these questions, a novel percutaneous self-expanding forceful reduction screw system was developed. The percutaneous transpedicular screw of this system is uniaxial with 0°, 3°, 6°, or 9° reduction angles. The integrated reduction angles of the screw can reinforce the distraction and lordosis for the fracture treatment, which is of great clinical value because pedicle screws are part of the fixation system that allows distraction and lordosis forces to be applied; hence, they are useful beyond their purpose of stabilization [26]. This reinforced distraction of this novel screw is and essential distinction from the conventional percutaneous transpedicular screw. The conventional type has a limited external distraction, and the limited distraction force was a parallel distraction of the posterior structures of the vertebral body, which could result in the partial extension of the intervertebral disc with the sacrifice of fracture reduction. This would eventually lead to the inadequate fracture reduction and internal fixation failure [27]. On the other hand, the novel percutaneous screw allows for the posterior column of the vertebral body to remain unchanged during the screw tightening process. Only the anterior column of vertebrae is extended to ensure the reduction of fracture and create the forces for lordosis; all of these features greatly improve the kyphosis caused by fracture. In the current report, the 38 patients with thoracolumbar fracture with severe vertebral compression were treated with the novel screw, and significant vertebral height restoration and kyphosis correction were achieved.

Another major issue with percutaneous fixation is progressive loosening when fusion is not performed concurrently. Given that the loosening of internal fixation is usually ascribed to the cap dropping, the drop-prevention bolt was developed along with the screw to avoid treatment failure due to internal fixation loosening. There was no cap dropping, internal fixation loosening, or failure among any of the 38 patients, indicating the effectiveness of this design.

## Conclusions

After the 2-year follow-up period of this study, the novel, percutaneous, self-expanding, forceful reduction screw system developed by us not only successfully expanded the minimally invasive percutaneous surgery to the thoracolumbar fracture with severe vertebral height loss but also achieved significant vertebral height restoration and kyphosis correction, as well as avoided the defects of conventional minimally invasive percutaneous surgery. However, further randomized clinical trials are needed to evaluate its advantage over the conventional open surgery for the treatment of this type of thoracolumbar fracture.
